# Cranial irradiation at early postnatal age impairs stroke-induced neural stem/progenitor cell response in the adult brain

**DOI:** 10.1038/s41598-020-69266-7

**Published:** 2020-07-23

**Authors:** Susanne Neumann, Michelle J. Porritt, Ahmed M. Osman, H. Georg Kuhn

**Affiliations:** 1 Department of Clinical Neuroscience, Institute for Neuroscience and Physiology, University of Gothenburg, Box 436, 405 30 Gothenburg, Sweden; 2 Department of Clinical Neuroscience, Center for Molecular Medicine, Karolinska Institutet, 171 76 Stockholm, Sweden; 3 Department of Women’s and Children’s Health, Karolinska Institutet, 171 64 Stockholm, Sweden

**Keywords:** Development of the nervous system, Neurogenesis, Regeneration and repair in the nervous system, Stem cells in the nervous system, Neural stem cells, Radiotherapy

## Abstract

Cranial irradiation (IR) is commonly used to treat primary brain tumors and metastatic diseases. However, cranial IR-treated patients often develop vascular abnormalities later in life that increase their risk for cerebral ischemia. Studies in rodents have demonstrated that IR impairs maintenance of the neural stem/precursor cell (NSPC) pool and depletes neurogenesis. We and others have previously shown that stroke triggers NSPC proliferation in the subventricular zone and migration towards the stroke-injured neocortex. Whether this response is sustained in the irradiated brain remains unknown. Here, we demonstrate that cranial IR in mice at an early postnatal age significantly reduced the number to neuronal progenitors responding to cortical stroke in adults. This was accompanied by a reduced number of microglia/macrophages in the peri-infarct cortex; however, the astrocytic response was not altered. Our findings indicate that IR impairs the endogenous repair capacity in the brain in response to stroke, hence pointing to another side effect of cranial radiotherapy which requires further attention.

## Introduction

Cranial irradiation (IR) is a routine clinical treatment for primary brain tumors and metastasis, it however causes long-lasting side effects in cancer survivors such as neurocognitive sequelae^1–4^. This has been particularly problematic in pediatric patients, as their brain is still developing^5^. Nowadays, irradiation of the brain in pediatric patients is largely abandoned or delayed until the neuronal maturation. Possible mechanisms for cognitive effect have been suggested to be related to depletion of postnatal and juvenile neurogenesis^6^ and the development of cerebrovascular complications, and even stroke in later life^7^, which collectively leads to poor quality of life for the cancer survivors^8–10^.

In the CNS of most mammals, neurogenesis exists throughout life. Neural stem/precursor cells (NSPC) reside in discrete regions e.g. the subventricular zone (SVZ) of the lateral ventricle and other areas along the ventricular system^11,12^. In the mouse SVZ, NSPCs give rise to neuroblasts that migrate to the olfactory bulb where they differentiate into neurons that integrate into the pre-existing neuronal networks^13,14^. NSPCs are highly proliferative and therefore are susceptible to DNA damage caused by IR leading to cell death^15,16^. In experimental animal models, moderate doses of IR lead to a long-lasting decline in NSPC proliferation and neurogenesis in a dose- and age-dependent manner^17–20^.

On the other hand, cerebral ischemia increases NSPC proliferation in the SVZ and re-routes migration of the neuronal progenitors towards the injury site^21–26^. Whether this progenitor cell response to stroke is affected by IR remains unknown. In this study, we investigated the NSPC response to cortical stroke in the adult mouse brain after exposure to IR at an early postnatal stage. We assessed NSPCs migration and neuroinflammation in the peri-infarct area, and we demonstrate that IR significantly diminishes stroke-induced NSPC response and accumulation of microglia/macrophages (MG/MQs) in the peri-infarct area.

## Materials and methods

### Animals

**Figure 1:**
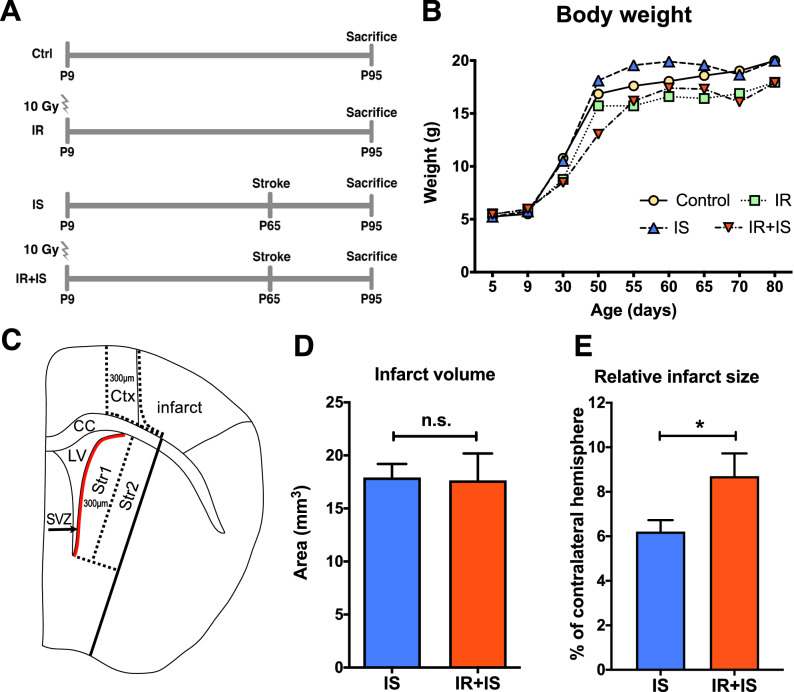
Experimental design, measurement of the body weight and infarct size. (**A**) Experimental design. Animals were irradiated with 10 Gy on postnatal day 9 (P9) and a photo-thrombotic stroke was induced on P65. Animals where sacrificed 30 days after stroke (P95). Experimental groups noted as: Ctrl = Control; IR = Irradiation; IS = Stroke; IR + IS = Irradiation + Stroke. (**B**) Body weight was determined in regular intervals throughout the experimental period. Control: Sham-irradiated, sham-stroke lesioned animals, IR: Irradiated, sham-stroke lesioned animals, IS: Sham-irradiated and stroke lesioned animals, IR + IS Irradiated and stroke lesioned animals (n = 13–16 per group). (**C**) Illustration depicts areas of histological quantification. Ctx: Peri-infarct cortex: Str1: Striatum 1 (closest to the SVZ); Str2: Striatum 2 (in 300 µm distance from the SVZ); CC: Corpus callosum; LV = lateral ventricle. (**D**) Measurement of the cortical infarct volume in mm^3^ (n = 11 for IS and IR + IS). (**E**) Measurement of the relative infarct size in percent of the contralateral hemisphere. (n = 11 for IS and IR + IS).

Female C57Bl/6N pups (Charles River, Germany) were initially housed with their dam and after weaning group housed (n = 6 per cage) in individually ventilated cages under equal light/dark cycle with ad libitum access to food and water. Experimental procedures were performed in accordance to the Swedish and European animal welfare regulations and approved by the Gothenburg committee of Swedish Animal Welfare (application: 317-2012). On postnatal day 5 (P5), when littermate pups were delivered from the breeding facility, 68 mice were randomly assigned to four experimental groups (each n = 17): Control animals that were only subjected to anesthesia (Ctrl), irradiation only (IR), cortical ischemia only (IS) and mice exposed to both irradiation and cortical ischemia (IR + IS) (Fig. 1A). Four pups died before P9, four mice did not wake up from anesthesia during the irradiation procedure on P9. Mice were weaned on P21. Four additional animals died after the stroke procedure on P65, leaving a total number of mice for morphological analysis as follows: Ctrl n = 15, IR n = 12, IS n = 15, IR + IS n = 14.

### Irradiation

Pups were subjected to cranial irradiation on postnatal day 9 (P9). Under anesthesia with an intraperitoneal injection of xylazine (20 mg/ml; Rompun, Bayer Healthcare AG) and ketamine (50 mg/ml; Ketalar, Pfizer), mice were placed on a polystyrene bed in a prone position. The animal’s head was covered with a 15 mm bolus of solid tissue-equivalent gel. Pups received a single dose of 10 Gy irradiation using a linear accelerator (Varian Clinac 600CD, Radiation Oncology Systems LLC, San Diego, CA, USA) with 6MV nominal photon energy and a dose rate of 2.08 Gy/min (Dose variation of ± 5%). A symmetrical 2 × 2 cm radiation field was used for whole head irradiation. The source to skin distance was adjusted for approximately 100 cm. Control animals received the same dosage of anesthesia. All animals were allowed to recover from the anesthesia before they were returned to their biological dams.

### Cortical stroke

Photothrombotic cortical ischemia was performed on P65 as previously described^26,27^. Briefly, mice were anesthetized with isoflurane (5% induction and 1.5% maintenance; Isobavet; Schering-Plough Corporation, Kenilworth, NJ) in a mixture of air and oxygen (1:1) and sustained with 2.5% isoflurane during surgery. Mice were placed in a stereotaxic frame and body temperature was maintained using a heating pad. A scalp incision was made, the skin retracted to expose the skull, and the periosteum removed to allow exposure of the skull. Rose Bengal dye (0.1 ml of 10 mg/ml) was injected intraperitoneally 5 min prior to laser illumination. The laser source (https://www.coboltlasers.com/lasers/dpss-lasers: 25–500 mW, cw single-frequency laser, linewidth < 1 MHz) was placed 3 cm above the skull. The skull and underlying tissue were illuminated for 10 min with a laser beam (power; 50 mW, wavelength; 561 nm) at the coordinates relative to bregma: + 2.4 mm lateral and 0.5 mm anterior. After illumination, the skin was sutured, and animals placed in a heated box until they recovered. Non-ischemic control animals where treated identically with respect to Rose Bengal injection, anesthesia, scalp incision and suture. Only the laser beam illumination was not performed.

### Tissue preparation

Thirty days post-stroke, animals were deeply anesthetized with sodium pentobarbital, transcardially perfused with 0.9% sodium chloride, followed by 4% paraformaldehyde in 0.1 M phosphate buffer (pH 7.4). Brains were collected and post-fixed in the same fixative for 24 h, then transferred to 30% sucrose in 0.1 M phosphate buffer for cryoprotection and left for a minimum of 3 days. Brains were cryosectioned coronally into 25 µm free-floating sections using a sliding microtome (Leica SM2000R) and stored as 1:12 series at 4 °C in tubes containing cryoprotection solution (25% glycerin, 25% ethylene glycol in 0.1 M phosphate buffer) for further histological analysis.

### Immunohistochemistry and immunofluorescence

Sections were incubated in sodium citrate pH 6.0 for 30 min at 80 °C when antigen retrieval was needed. When the immunoperoxidase method was used, the endogenous peroxidase was quenched by incubating sections in 0.6% H_2_O_2_ for 30 min at room temperature. Non-specific binding was blocked by incubating sections in a solution of 3% normal donkey serum (Jackson ImmunoResearch Laboratories, West Grove, PA), 0.1% Triton X-100 in TBS for 30 min at room temperature. Sections were incubated at 4 °C for 24–48 h with the primary antibodies. The following primary antibodies were used: goat anti-DCX (1:100, Santa Cruz Biotechnology, Santa Cruz, CA, #sc-8066); rabbit anti-Iba1 (1:1,000; Wako Pure Chemical Industries Ltd., #01919741), rat anti-CD68 (1:500; Nordic BioSite, #MCA1957T) or rabbit anti-GFAP (1:1,000; Dako, #z0334). After rinsing in TBS, the sections were incubated followed by 1 h or 2 h incubation with appropriate biotinylated or fluorescent secondary antibodies, respectively. The following secondary antibodies were used: biotinylated donkey anti-goat IgG (1:1,000, Jackson ImmunoResearch Lab, #705-065-147); donkey anti rabbit-Alexa 555 (#A31572), donkey anti rat-Alexa 488 (#A21206), or donkey anti rabbit-Alexa 488 (#A21208, all at 1:1,000; Invitrogen Molecular Probes). ToPro3 (1:1,000; Molecular Probes/Life Technologies) was applied as nuclear counterstain for immunofluorescence procedures. To visualize the immunoperoxidase staining, sections were incubated for 1 h in avidin–biotin solution (1:100; Vectastain ABC Elite kit, Vector Laboratories, Burlingame, CA). The color precipitate was developed with H_2_O_2_, nickel chloride and 3–3′diaminobenzidine tetrahydrochloride (DAB; 1:100; Saveen Werner AB, Malmö, Sweden). Sections were mounted onto glass slides and coverslipped using NeoClear and NeoMount (Merck, Whitehouse Station, NJ) for immunoperoxidase staining or ProLong Gold anti-fade reagent with DAPI (Molecular probes/Life technologies) for the fluorescent staining.

### Histological analysis

Analysis was performed by an experimenter blinded to treatment. No animals were excluded from the current experiment; however, some sections reaching into the corpus callosum were exchanged for neighboring sections without corpus callosum damage, when clear delineation of lesion border was necessary for analysis. Some animals were excluded from individual histological analyses, if the immunostaining procedures generated insufficient staining quality to differentiate between immune signal and background, while others were excluded when tissue fragility led to section loss during the free-floating staining procedures. The specific animal numbers for each analysis are reported in the figure legends. Analyses were performed on all animals, except for analysis of astrocyte (GFAP intensity) and microglia (IBA1/CD68) activation, where randomly 8 animals per group were chosen for analysis.

The infarct volume was determined by outlining the area of necrotic core on every 6th tissue section. Infarct volume was calculated by multiplying the sum of the areas by the tissue thickness (25 µm) and series interval (6) and expressed as mm^3^. To quantify the density of labelled cells, three distinct areas within the ipsilateral hemisphere were delineated in Fig. 1C as previously described^26^: Peri-infarct cortex (Ctx; aligned to the infarct border and 300 µm peri-infarct), striatum 1 (Str1, area including the lateral ventricle wall up to 300 µm lateral into the striatum) and striatum 2 (Str2, adjacent to the lateral border of Str1).

**Figure 2:**
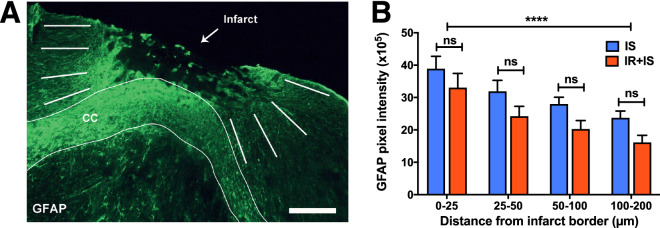
Irradiation did not affect the astrocytic reactivity in the ischemic cortex. (**A**) Representative fluorescent image of GFAP immune-reactivity in the peri-infarct area surrounding the lesion. It is also indicated where GFAP intensity was measured*. CC* Corpus callosum; scale bar = 1000 µm. (**B**) Bar graph of GFAP fluorescence intensity measurements in the per-infarct area. 2-way ANOVA with Bonferroni multiple comparison, IS = Stroke (n = 8) and IR + IS = Irradiation + Stroke (n = 8); ****p ≤ 0.0001 for distance effect).

**Figure 3:**
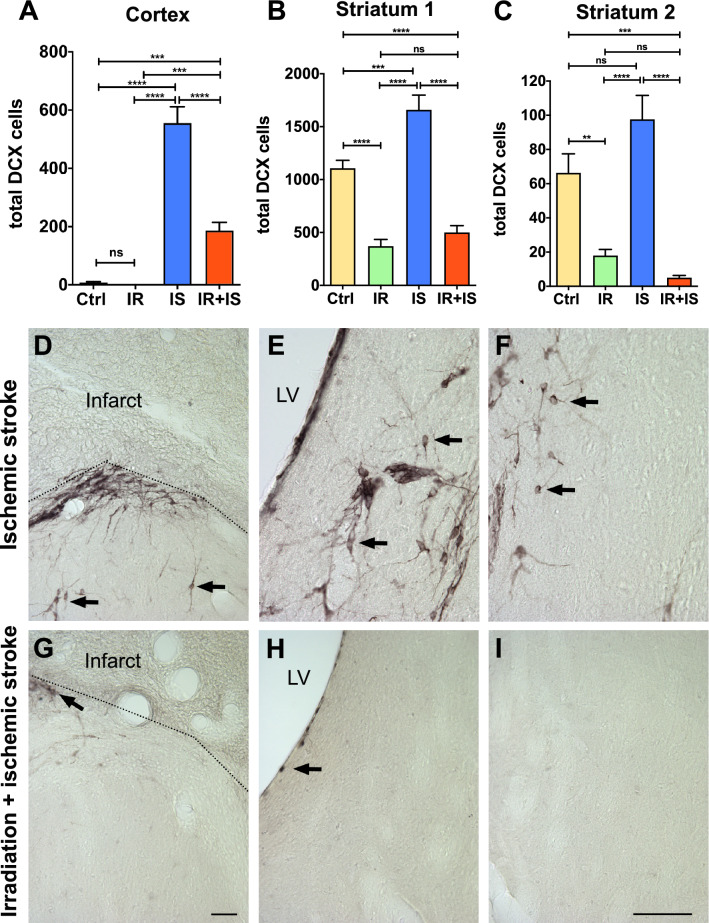
Irradiation reduced the number neuronal progenitors responding to cortical stroke. (**A**–**C**) Quantification of DCX^+^ neuronal progenitor cells in the cortex (**A**), two regions of the striatum, Striatum 1 adjacent to the SVZ (B), Striatum 2 lateral to Striatum 1 (**C**). One-way ANOVA with Bonferroni multiple comparisons test, Ctrl = Control (n = 15); IR = Irradiation (n = 10); IS = Stroke (n = 11) and IR + IS = Irradiation + Stroke (n = 13), **p ≤ 0.01; ***p ≤ 0.001; ****p ≤ 0.0001). (**D–I**) Representative images of DCX^+^ cells in IS (**D**–**F**) and IR + IS (**G**–**I**) brain regions. Cortex (**D**, **G**), Striatum 1 (**E**, **H**), Striatum 2 (**F**, **I**), scale bars = 100 µm.

The total numbers of DCX^+^ and Iba1^+^ cells were determined using a stereology system (Stereo Investigator; Micro-BrightField Inc). Optical fractionator was overlaid on the ipsilateral striatum and the peri-infarct area in every 6th section per animal covering the three defined regions (Fig. 1C; Ctx, Str1 & Str2). Cells were considered for quantification when the cell body was clearly identifiable. Quantification was performed for DCX cells in 8 and for Iba1 cells in 3 sections per animal, respectively. The activation state of MG/MQs was assessed by determining the percentage of Iba1^+^ cells co-expressing CD68 (Iba1 + /CD68 +) using a laser scanning confocal microscope (Leica TCS SP2; Wetzlar, Germany) using the following settings: magnification: 63x; resolution: 512 × 512; mode: 12/12 bit; exposure: 500, gain: 700; laser beam intensity: 488 nm—35%, 546 nm—70%, 633 nm: 50%; average: 4; scanning speed: 300 Hz, . The overview picture (Fig. 2A) and DCX staining (Fig. 3A, magnification 40x) were obtained using a light microscope (Leica DM6000, settings: Field 33, Aperture 38, Intensity 7–9). Iba1 + /CD68^+^ cells were counted along the infarct border in an area of 200 µm using a 63 × objective with sequential fluorochrome excitation at 488 nm, 546 nm and 633 nm. A minimum of 100 Iba1^+^ cells per animal were analyzed in 3 sections of eight randomly selected animals per group. The relative infarct size was determined (supplemental Fig. S1) using a Zeiss airyscan LSM 880 (magnification 10x) and the ZEN 2.1 SP3 FP3 software (*Settings:* frame: 512 × 512; line step: 1; speed: 7; average: 4; bit depth: 12; laser 488 nm: 3.0; gain: 750; tile scan: 8 × 11) by outlining the area of necrotic core using ImageJ (measure function, pixel-µm conversion with the set scale function based on scale bar in picture). Relative infarct size was calculated by dividing the sum of all measured necrotic cores per animal by the size of the sum of all measured (3 sections selected to represent different parts of the lesion) contralateral hemispheres per animal and multiplied by 100 giving the percentage of the brain size.

To determine the astrocytic reactivity, fluorescent intensity of GFAP immunoreactivity was measured in the peri-infarct cortex in eight randomly selected animals per group (three sections per animal) using ImageJ^28^. To capture the whole peri-infarct region 5–7 pictures where taken using a laser scanning confocal microscope (Leica TCS SP2; Wetzlar, Germany) using a 20 × objective with sequential fluorochrome excitation at 488 nm. The settings where maintained throughout all samples: magnification: 20x; resolution: 512 × 512; mode: 12/12 bit; exposure: 500; gain: 500; laser beam intensity: 488 nm—35%; average: 4; scanning speed: 400 Hz. In each section, the intensity of GFAP expression was assessed in eight areas along the infarct border spanning a distance of 200 μm from the infarct border divided into five zones based of proximity to the infarct border (0–25, 25–50, 50–100 and 100–200 μm; Fig. 2A). Intensity was measured using the line tool and the *Analyze Plot Profile* function in ImageJ, conversion pixel to µm was done based on the scale bar. The local background was then subtracted from measured fluorescent intensities. The fluorescent intensities acquired from the eight measuring areas of each proximal zone per section were averaged and fluorescent intensities from the three analyzed sections were summed and averaged. The overview picture presented in supplementary Fig. S2 was captured with the Zeiss airyscan LSM 880 (*Settings:* frame: 512 × 512; Line step: 1; speed: 5; average: 4; bit depth: 12; laser 488 nm: 2.8; gain: 750; tile scan: 5 × 8).

### Statistical analysis

All statistical analysis was performed using GraphPad Prism (version 8.31). Data are presented as mean ± standard error of the mean (SEM). Due to some missing values on postnatal day P50, P55, P60, animal weights were analyzed using a linear mixed effects model (REML, GraphPad Prism v8.31). Data on cell quantification were analyzed using one-way ANOVA unless only IS and IR + IS groups were compared using unpaired two-tailed t-test. GFAP intensity were analyzed using a two-way repeated measure ANOVA. Significance was assumed at *P* < 0.05 after adjustment using Bonferroni’s multiple comparisons post hoc test. Detailed ANOVA results are summarized in Supplementary Tables 1–7.

## Results

### Irradiation reduces weight gain but does not affect the infarct size after cortical ischemia

We first assessed the effect of IR and/or ischemia on weight gain by determining body weight at P5, P9 (the day of IR), P30, every 5 days between P50 and P70, as well as P80 (Fig. 1B). All groups displayed significant weight gain over time, groups differed significantly from each other except for controls vs. stroke and IR vs. IR&IS (mixed effect model, main group effects, all *p* < 0.001). At the time of irradiation on P9, we observed no differences between groups. At the time of stroke induction on P65, irradiated animals had a significantly reduced body weight compared to non-IR animals (mixed effect model, with Bonferroni correction, adjusted *p* < 0.005). After stroke, the significant difference in weight between irradiated and non-irradiated animals (irrespective, whether ischemic or non-ischemic) remained (mixed effect model with Bonferroni correction, all adjusted *p* < 0.001). Cortical ischemia (irrespective if irradiated or sham-irradiated) had no effect on body weight (Fig. 1B). For a complete statistical summary of body weight analysis see Supplementary Table S1.

We next evaluated the impact of IR on the stroke lesion and found the absolute size (infarct volume) of the lesion to be not different between the stroke-lesioned groups (Fig. 1D, unpaired two-tailed *t* test, *p* = 0.926, *t* = 0.094, df = 20, for images see supplemental Fig. S1). However, since the brain size was affected by irradiation, we found a significant difference in the infarct size relative to the size of the contralateral hemisphere in the ischemic stroke (IS) vs. irradiation + stroke (IR + IS) animals (Fig. 1E, unpaired two-tailed *t* test, *p* = 0.0425, t = 2.167, df = 20).

### Irradiation does not affect the astrocyte reactivity after cortical ischemia

After ischemia, astrocytes become reactive and upregulate the expression of glial fibrillary acidic protein (GFAP). Thus, we next evaluated the expression of GFAP in the injured cortex covering an area of 200 μm from infarct border (Fig. 2A). In both IR and non-IR animals after stroke, the expression of GFAP was highest close to the infarct border and significantly decreased with further distance from the lesion site (Fig. 2B, for images see Supplementary Fig. S2). Even though the average GFAP intensity for the IR + IS group was persistently below the average of the IS group, no significant difference in GFAP expression was detected between the groups at any distance (for 2-way ANOVA summary see Supplementary Table S2). These data suggest that IR did not affect stroke-induced GFAP-immunoreactivity of astrocytes.

### Irradiation diminishes the number of neuronal progenitors in the injured cortex

Cortical stroke is known to trigger migration of neuronal progenitors from the SVZ towards the injured cortex and the striatum^29–31^, therefore we next wanted to address whether IR affects this response. We quantified the number of neuronal progenitors (DCX^+^ cells) in the peri-infarct cortex, as well as in the ipsilateral striatum in two areas, close to the SVZ and distant from SVZ (Fig. 3A–C). Scarce DCX^+^ cells were observed in the cortex of the non-ischemic animals, however after cortical ischemia the number of DCX^+^ cells was drastically increased in the peri-infarct cortex (both IR and non-IR animals) compared to non-ischemic animals (Fig. 3A,D,G, for ANOVA summary see Supplementary Table S3). Comparison of the number of DCX^+^ cells in the peri-infarct cortex of the IR and non-IR ischemic animals, revealed that IR animals had significantly less DCX^+^ cells (Fig. 3A). In the striatum, irradiation decreased the number of DCX^+^ cells that are normally observed in the intact brain, and also diminished ischemia-induced increase in DCX^+^ cells numbers in the striatum (Fig. 3B,C,E,F,H,I, for ANOVA summary see Supplementary Table S4 and S5). These data suggest that IR compromises the neuronal response triggered by cortical stroke.

### Irradiation reduces the microglial/macrophage accumulation, but not the activation state, in response to cortical ischemia

**Figure 4:**
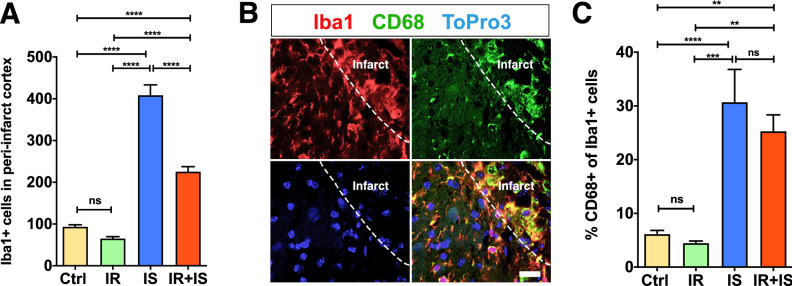
Irradiation reduced accumulation of microglia/macrophages in the ischemic cortex. (**A**) Bar graph shows quantification of Iba^+^ cells in the peri-infarct cortex (One-way ANOVA, multiple comparison, Ctrl, control; IR, Irradiation; IS, Stroke IR + IS = Irradiation + Stroke, all groups n = 8, **p ≤ 0.01; ***p ≤ 0.001; ****p ≤ 0.0001). (**B**, **C**) analysis of MG/MQ activation as evaluated by co-expression of Iba1 and CD68 (Iba1 + /CD68 +). (**B**) Confocal image displays expression Iba1 + (red) and CD68^+^ (green). ToPro3 nuclear counterstain (blue), merged Scale bar 20 μm. (**C**) Bar graph shows the percentage of Iba1^+^/CD68^+^ of Iba1^+^ cells in the peri-infarct cortex. (One-way ANOVA, multiple comparison, Ctrl = Control n = 15; IR = Irradiation n = 10; IS = Stroke n = 11 and IR + IS = Irradiation + Stroke n = 13, **p ≤ 0.01; ***p ≤ 0.001; ****p ≤ 0.0001).

After brain injury, MG/MQ accumulate at the injury site and acquire an activation state. Therefore, we next analyzed the response of MG/MQ in the neocortex as judged by the expression of Iba1 (Iba1^+^). Ischemic animals (both irradiated and non-irradiated) had a significant increase in the number of Iba1^+^ cells compared to non-ischemic animals (Fig. 4A, for ANOVA summary see Supplementary Table 6). When we compared the effect of the IR on the MG/MQ accumulation after cortical ischemia, we found that IR significantly decreased Iba1^+^ cells accumulating in the injured cortex (Fig. 4A).

We next assessed the MG/MQ activation state based on the expression of CD68 among the Iba1-positive cells (CD68^+^/Iba1^+^, see Fig. 4B). Ischemic animals had a significant increase in the percentage of Iba1^+^ cells co-expressing CD68^+^ compared to non-ischemic animals, however, we did not detect a difference in the percentage of Iba1^+^/CD68^+^ cells between the IR and non-IR animals after ischemia (Fig. 4C, for ANOVA summary see Supplementary Table 7). Together, these data indicate that IR prior cortical ischemia weakens the ischemia-induced MG/MQ accumulation in the injured cortex, but not their activation.

## Discussion

Whole brain irradiation decreases NSPC proliferation and diminishes neurogenesis, not only in the hippocampus but also in the subventricular zone^32–35^. It is also established in rodents that NSPCs respond to damage, such as stroke, and contribute to the repair process that is critical for post-stroke functional recovery^36^. We have previously shown^33,34^ that the response to postnatal irradiation is niche-dependent, i.e. with a lack of recovery in the hippocampus, while the SVZ showed not full, but substantial recovery of stem cell activity. Here, we reveal that even with potential recovery of stem cell activity in the SVZ after IR at an early developmental stage, the NSPC response to cortical ischemia in adulthood remains compromised.

NSPC response to injury is regulated by a number of factors, including inflammatory signals^37–39^. Microglia/macrophages, the resident immune cells in the brain, proliferate after stroke, accumulate at the injury site^40–42^, and secrete a number of inflammatory mediators such as chemokines and cytokines that increase NSPC proliferation and instruct migration towards the injury site^43,44^. It has also been shown that MG/MQ are susceptible to damage by IR, especially at early postnatal ages, leading to decreased MG/MQ numbers^45^. Decreased numbers of MG/MQ in response to IR in all, not only neurogenic active areas of the brain^45^. This was attributed to MG/MQ being injured and dying after IR. The effect of IR, which was age-dependent, can be explained by MG/MQ being highly proliferative between P5 and P14^46^ making them especially vulnerable to radiation-induced damage.

In this study we show that a dose of 10 Gy IR delivered to the mouse brain at postnatal day 9 decreased ischemia-induced accumulation of MG/MQ in the adult peri-infarct cortex, which is indicative of a modest inflammatory response to the cortical ischemia after IR. The reduced number of neuronal progenitors detected in the peri-infarct cortex after IR could thus be either directly the result of IR-induced reduction of the NSPC pool in the SVZ^16,47,48^, changed vascularization^49,50^ or indirectly the effect of diminished inflammatory signals from MG/MQ that serve as chemoattractant for migration of neuronal progenitors towards the injured cortex^29^ or a combination of those factors.

The infarct area in % of the contralateral hemisphere, here called relative infarct area, was significantly bigger in IR + IS animals than in IS alone. However, we could not observe a difference in absolute infarct volume, indicating a smaller overall brain size. In our study, IR + IS animals are significantly lighter than IS animals and a negative impact of irradiation on weight gain has previously been reported in rodents^51^ and in head and neck cancer patients which are affected by significant weight loss over time^52^. This is especially true in female mice as they are more susceptible to irradiation-induced brain size reduction^35,53^. Further, an increased injury size after IR has previously been reported by Zhu et al.^54^ using a hypoxia–ischemia brain injury model. The observed body weight differences might display growth delay of the brain and the body in the juvenile phase induced by irradiation. The growth later on (after P50) follows the same pattern in all groups, with just a lower starting point in the IR and IR + IS group.

We measured the number of DCX^+^ cells, which represent late progenitors, neuroblasts as well as young neurons. IR-treated animals showed decreased numbers of DCX^+^ cells in the striatum as well as in the peri-infarct cortex. After ischemic stroke, both groups showed an increase of DCX^+^ cells in the peri-infarct region, although the stroke response in the irradiation-treated group (IR + IS) was not as pronounced as in the non-irradiated animals. It is for us not possible to say if these observations are exclusively based on the depleted pool of stem cells or if IR also effects proper maturation and/or migration of DCX^+^ cells. In this study, we have not addressed if neural progenitor cells continue to develop into mature neurons, since we have previously shown that maturation of neurons in the stroke-lesioned cortex is extremely limited^26^. However, it is important to confirm that despite of a compromised SVZ stem cell pool, a substantial response of neural progenitor cells to stroke is still detectable even after brain irradiation at 10 Gy.

Astrocytes are important cellular components in the ischemic cortex and crucial for regulating the infarct size by forming the glial scar^55^. The glial scar arises from regional astrocytes, already present in the area where the ischemic insult occurred^56^, and also from newly generated astrocytes derived from NSPC that migrate from the SVZ in response to injury^57^. It has been reported that astrocytes react with gliosis to irradiation itself^58,59^. In this study, we show that the astrocytic response to adult cortical stroke was not affected by IR at postnatal day 9, however, there is a tendency of a decreased astrocyte response in IR + IS animals. This may hint to an impaired maturation GFAP + astrocytes after IR treatment or IR-induced astrocyte senescence^60^. Alternatively, due to the lower numbers of MG/MQ, signals released by MG/MQ to initiate an astrocyte response (i.e. cytokines) are less effective. Whether the NSPCs from SVZ may contribute to the formation of the glial scar after IR remains unclear and requires further investigation.

Overall, IR-impaired ischemia-induced neuronal progenitor response may have clinical significance, as pediatric cancer survivors are subjected to IR and are prone to develop neurovascular diseases, such as ischemic stroke^61–63^, and the compromised endogenous repair capacity may worsen outcome after a secondary brain injury^64^. A deeper understanding of the mechanisms of IR-induced impairment in the NSPC response to brain injury would be valuable to develop interventions to prevent the loss or enhance the endogenous regeneration potential after brain injuries in cancer survivors^65^.

## Supplementary information

Supplementary Information.
