# Supplementary material for: Cranial irradiation at early postnatal age impairs stroke-induced neural stem/progenitor cell response in the adult brain
Source: Sci Rep. 2020 Jul 23;10:12369. doi: 10.1038/s41598-020-69266-7 (PMC7378832; doi:10.1038/s41598-020-69266-7)
Supplement: Supplementary file 1 — Supplementary Information. [file 41598_2020_69266_MOESM1_ESM.pdf]

**Supplementary Information**

**Cranial Irradiation at Early Postnatal Age Impairs Stroke-induced Neural Stem/Progenitor Cell Response in the Adult Brain**

Susanne Neumann, Michelle J. Porritt, Ahmed M. Osman, H. Georg Kuhn

**Supplementary Figure S1**

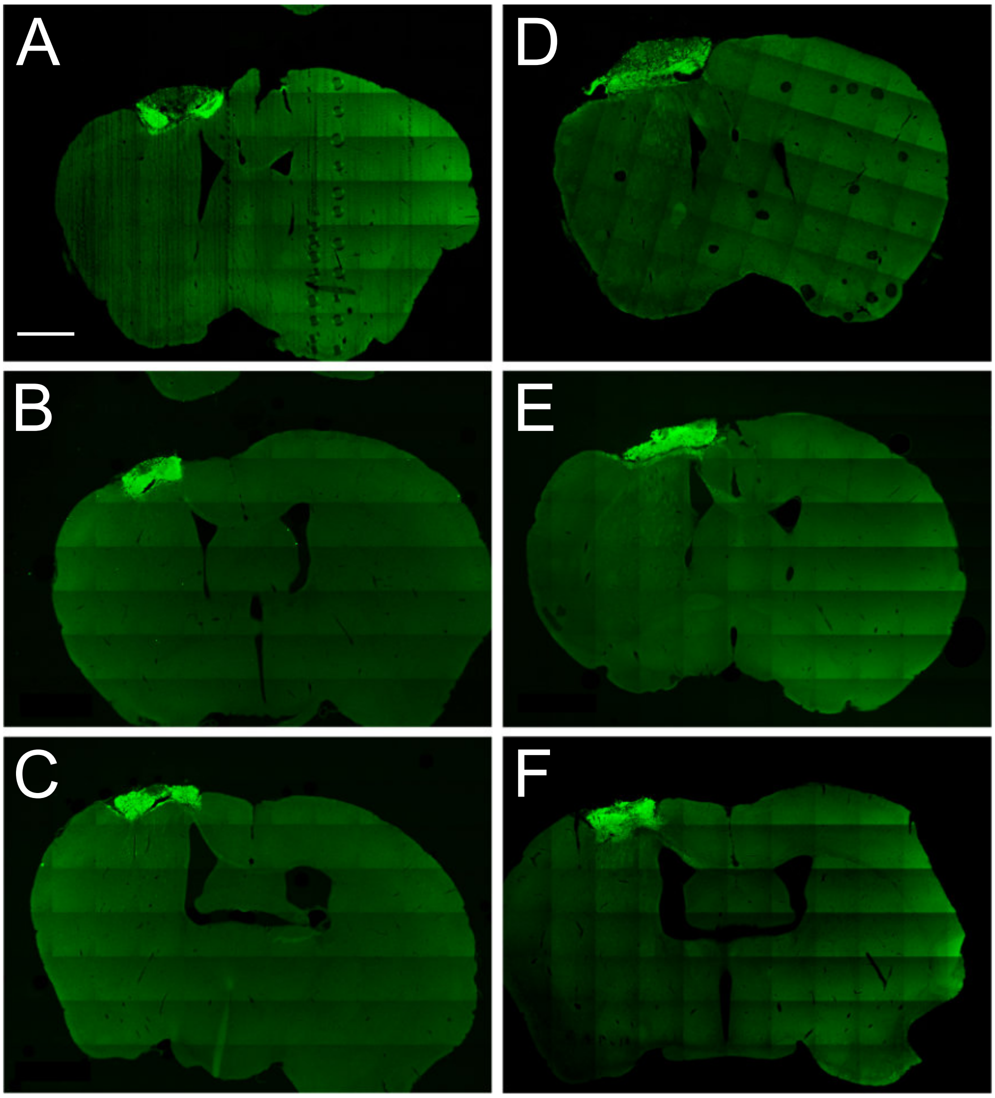


**Supplemental Figure S1: Infarct size. (A)-(C)** Representative images of the infarct area in non-irradiated animals (IR+IS) and **(D)-(F)** irradiated animals (IS). Overview images taken with the Zeiss airyscan LSM 880 (magnification 10x) using the ZEN 2.1 SP3 FP3 software (*Settings:* Frame: 512x512; Line step: 1; speed: 8; Average: 4; Bit depth: 12; laser 488 nm: 2.8; Gain: 800; Tile scan; 8x11), Scale bar in A = 1mm.

**Supplementary Figure S2**

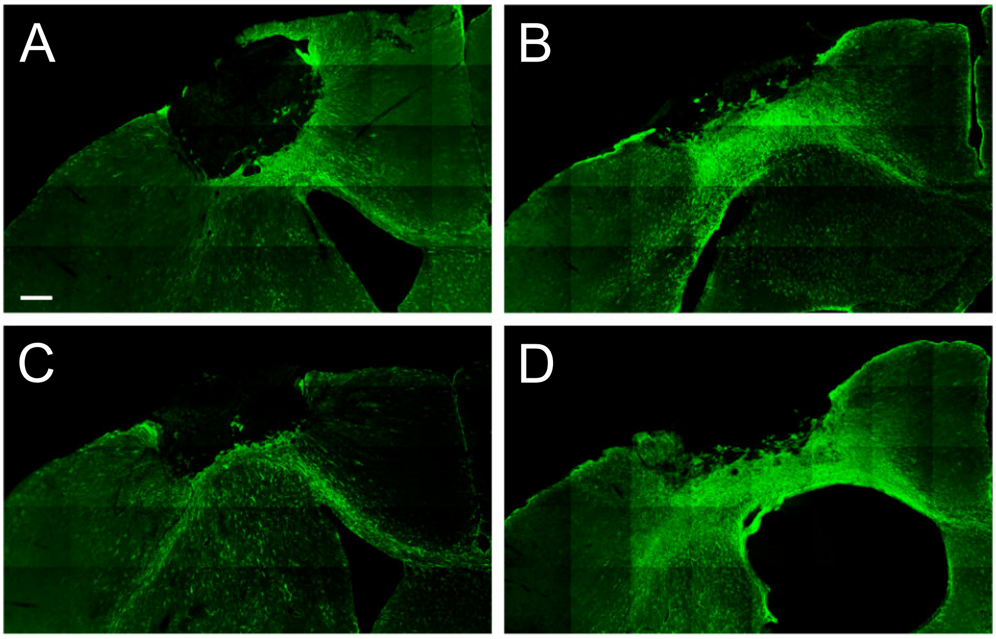


**Supplementary Figure S2: A comparison between two different spots within one lesion of each condition.** For measuring GFAP activity in the area surrounding the infarct, sections from the middle infarct area were selected. The lesion area varies depending on the distance of the section from the center of the lesion (**A,C**: outside the lesion center, **B,D**: lesion center). Differences between the groups **(A, B:** IS - ischemic stroke) and **(C, D:** IR+IS - irradiation + ischemic stroke) where not apparent. Overviews were taken with the Zeiss airyscan LSM 880 (magnification 20x) using the ZEN 2.1 SP3 FP3 software (*Settings:* Frame: 512x512; Line step: 1; speed: 5; Average: 4; Bit depth: 12; laser 488 nm: 2.8; Gain: 750; Tile scan; 5x8), Scale bar in A = 250 µm.

# Supplementary Table S1: Statistical summary of body weight

| | | | | | | | | |
|---|---|---|---|---|---|---|---|---|
| **Body weight** | | | | | | | | |
| **Mixed-effects model (REML)** | Matching: Stacked | | | | | | | |
| Assume sphericity? | No | | | | | | | |
| Alpha | 0.05 | | | | | | | |
| | | | | | | | | |
| **Fixed effects (type III)** | **P value** | **P value summary** | **significant** | **F (DFn, DFd)** | | **Geisser-Greenhouse's epsilon** | | |
| Time | <0.0001 | **** | Yes | F (2.473, 111.3) = 2197 | | 0.3092 | | |
| Column Factor | <0.0001 | **** | Yes | F (3, 59) = 26.08 | | | | |
| Time x Column Factor | <0.0001 | **** | Yes | F (24, 360) = 13.73 | | | | |
| | | | | | | | | |
| Random effects | SD | Variance | | | | | | |
| Subject | 0.7773 | 0.6042 | | | | | | |
| Residual | 0.8727 | 0.7616 | | | | | | |
| | | | | | | | | |
| Was the matching effective? | | | | | | | | |
| Chi-square, df | 143.3, 1 | | | | | | | |
| P value | <0.0001 | | | | | | | |
| P value summary | **** | | | | | | | |
| Is there significant matching? | Yes | | | | | | | |
| | | | | | | | | |
| **Bonferroni's multiple comparisons test** | **Predicted (LS) mean diff.** | **95.00% CI of diff.** | **Significant?** | **Summary** | **Adjusted P Value** | | | |
| Control vs. IR | 1.469 | 0.5879 to 2.349 | Yes | *** | 0.0002 | | | |
| Control vs. IS | -0.5048 | -1.319 to 0.3093 | No | ns | 0.5745 | | | |
| Control vs. IR+IS | 1.709 | 0.8970 to 2.522 | Yes | **** | <0.0001 | | | |
| IR vs. IS | -1.973 | -2.840 to -1.106 | Yes | **** | <0.0001 | | | |
| IR vs. IR+IS | 0.2407 | -0.6245 to 1.106 | No | ns | >0.9999 | | | |
| IS vs. IR+IS | 2.214 | 1.417 to 3.011 | Yes | **** | <0.0001 | | | |

Body weight - analysis of individual days

| **Bonferroni's multiple comparisons test** | **Mean Diff.** | **95.00% CI of diff.** | **Significant?** | **Summary** | **Adjusted P Value** | | | |
|---|---|---|---|---|---|---|---|---|
| **Day 5** | | | | | | | | |
| Control vs. IR | -0.2716 | -0.8255 to 0.2822 | No | ns | >0.9999 | | | |
| Control vs. IS | -0.003309 | -0.6260 to 0.6194 | No | ns | >0.9999 | | | |
| Control vs. IR+IS | -0.2092 | -0.7899 to 0.3715 | No | ns | >0.9999 | | | |
| IR vs. IS | 0.2683 | -0.3274 to 0.8640 | No | ns | >0.9999 | | | |
| IR vs. IR+IS | 0.06244 | -0.4883 to 0.6132 | No | ns | >0.9999 | | | |
| IS vs. IR+IS | -0.2059 | -0.8262 to 0.4145 | No | ns | >0.9999 | | | |
| **Day 9** | | | | | | | | |
| Control vs. IR | -0.3385 | -0.8487 to 0.1718 | No | ns | 0.4185 | | | |
| Control vs. IS | -0.2412 | -0.7919 to 0.3096 | No | ns | >0.9999 | | | |
| Control vs. IR+IS | -0.4647 | -0.9229 to -0.00647 | Yes | * | 0.0454 | | | |
| IR vs. IS | 0.09729 | -0.4235 to 0.6181 | No | ns | >0.9999 | | | |
| IR vs. IR+IS | -0.1262 | -0.5454 to 0.2929 | No | ns | >0.9999 | | | |
| IS vs. IR+IS | -0.2235 | -0.6939 to 0.2469 | No | ns | >0.9999 | | | |
| **Day 30** | | | | | | | | |
| Control vs. IR | 1.995 | 0.9354 to 3.055 | Yes | *** | 0.0003 | | | |
| Control vs. IS | 0.2485 | -0.7201 to 1.217 | No | ns | >0.9999 | | | |
| Control vs. IR+IS | 2.283 | 1.279 to 3.288 | Yes | **** | <0.0001 | | | |
| IR vs. IS | -1.747 | -2.536 to -0.9576 | Yes | *** | 0.0001 | | | |
| IR vs. IR+IS | 0.2881 | -0.5512 to 1.127 | No | ns | >0.9999 | | | |
| IS vs. IR+IS | 2.035 | 1.383 to 2.687 | Yes | **** | <0.0001 | | | |
| **Day 50** | | | | | | | | |
| Control vs. IR | 1.128 | -1.379 to 3.635 | No | ns | >0.9999 | | | |
| Control vs. IS | -1.264 | -3.607 to 1.078 | No | ns | 0.6508 | | | |
| Control vs. IR+IS | 3.813 | 0.8321 to 6.793 | Yes | ** | 0.0079 | | | |
| IR vs. IS | -2.393 | -4.089 to -0.6967 | Yes | ** | 0.0058 | | | |
| IR vs. IR+IS | 2.685 | 0.04791 to 5.321 | Yes | * | 0.0446 | | | |
| IS vs. IR+IS | 5.077 | 2.613 to 7.541 | Yes | *** | 0.0001 | | | |
| **Day 55** | | | | | | | | |
| Control vs. IR | 1.875 | -0.8196 to 4.569 | No | ns | 0.2971 | | | |
| Control vs. IS | -1.963 | -4.523 to 0.5972 | No | ns | 0.1821 | | | |
| Control vs. IR+IS | 1.436 | -1.205 to 4.077 | No | ns | 0.6915 | | | |
| IR vs. IS | -3.838 | -5.527 to -2.150 | Yes | *** | 0.0002 | | | |
| IR vs. IR+IS | -0.4388 | -2.316 to 1.438 | No | ns | >0.9999 | | | |
| IS vs. IR+IS | 3.399 | 1.953 to 4.845 | Yes | **** | <0.0001 | | | |
| **Day 60** | | | | | | | | |
| Control vs. IR | 1.439 | -1.345 to 4.223 | No | ns | 0.7144 | | | |
| Control vs. IS | -1.865 | -4.652 to 0.9215 | No | ns | 0.3204 | | | |
| Control vs. IR+IS | 0.6381 | -2.172 to 3.448 | No | ns | >0.9999 | | | |
| IR vs. IS | -3.304 | -4.482 to -2.127 | Yes | **** | <0.0001 | | | |
| IR vs. IR+IS | -0.8013 | -2.144 to 0.5418 | No | ns | 0.5569 | | | |
| IS vs. IR+IS | 2.503 | 1.120 to 3.886 | Yes | *** | 0.0002 | | | |

## Supplementary Table S1: Body weight - analysis of individual days, continued

| | | | | | | | | |
|---|---|---|---|---|---|---|---|---|
| **Day 65** | | | | | | | | |
| Control vs. IR | 2.16 | 0.5616 to 3.758 | Yes | ** | 0.0046 | | | |
| Control vs. IS | -1.005 | -2.621 to 0.6105 | No | ns | 0.5114 | | | |
| Control vs. IR+IS | 1.274 | -0.3955 to 2.943 | No | ns | 0.2302 | | | |
| IR vs. IS | -3.165 | -4.140 to -2.190 | Yes | **** | <0.0001 | | | |
| IR vs. IR+IS | -0.886 | -1.970 to 0.1979 | No | ns | 0.1661 | | | |
| IS vs. IR+IS | 2.279 | 1.166 to 3.391 | Yes | **** | <0.0001 | | | |
| **Day 70** | | | | | | | | |
| Control vs. IR | 2.149 | 0.9858 to 3.311 | Yes | *** | 0.0001 | | | |
| Control vs. IS | 0.3547 | -0.8290 to 1.538 | No | ns | >0.9999 | | | |
| Control vs. IR+IS | 2.956 | 1.326 to 4.585 | Yes | *** | 0.0002 | | | |
| IR vs. IS | -1.794 | -2.912 to -0.6752 | Yes | *** | 0.0006 | | | |
| IR vs. IR+IS | 0.8073 | -0.7833 to 2.398 | No | ns | 0.9195 | | | |
| IS vs. IR+IS | 2.601 | 0.9966 to 4.206 | Yes | *** | 0.0007 | | | |
| **Day 80** | | | | | | | | |
| Control vs. IR | 2.042 | 0.9206 to 3.162 | Yes | *** | 0.0001 | | | |
| Control vs. IS | 0.002667 | -1.068 to 1.073 | No | ns | >0.9999 | | | |
| Control vs. IR+IS | 2.086 | 0.7335 to 3.438 | Yes | ** | 0.001 | | | |
| IR vs. IS | -2.039 | -3.060 to -1.017 | Yes | **** | <0.0001 | | | |
| IR vs. IR+IS | 0.0444 | -1.273 to 1.362 | No | ns | >0.9999 | | | |
| IS vs. IR+IS | 2.083 | 0.8026 to 3.364 | Yes | *** | 0.0006 | | | |

Ctrl = control group, IR = irradiation, IS = ischemic stroke, IR+IS = irradiation+stroke

## Supplementary Table S2: Statistical summary of GFAP intensity

| | | | | | |
|---|---|---|---|---|---|
| Table Analyzed | GFAP intensity | | | | |
| | | | | | |
| Two-way RM ANOVA | Matching: Stacked | | | | |
| Assume sphericity? | No | | | | |
| Alpha | 0.05 | | | | |
| | | | | | |
| **Source of Variation** | **% of total variation** | **P value** | **P value summary** | **Significant?** | |
| Distance x Treatment | 0.1384 | 0.9179 | ns | No | |
| Distance | 30.77 | <0.0001 | **** | Yes | |
| Treatment | 11.41 | 0.0838 | ns | No | |
| Subject | 46.09 | <0.0001 | **** | Yes | |
| | | | | | |
| **ANOVA table** | **SS** | **DF** | **MS** | **F (DFn, DFd)** | **P value** |
| Distance x Treatment | 10.06 | 3 | 3.352 | F (3, 42) = 0.1673 | P=0.9179 |
| Distance | 2237 | 3 | 745.6 | F (3, 42) = 37.20 | P<0.0001 |
| Treatment | 829.5 | 1 | 829.5 | F (1, 14) = 3.466 | P=0.0838 |
| Subject | 3350 | 14 | 239.3 | F (14, 42) = 11.94 | P<0.0001 |
| Residual | 841.8 | 42 | 20.04 | | |
| | | | | | |
| **Difference between column means** | | | | | |
| Mean of IS | 30.63 | | | | |
| Mean of IR+IS | 23.43 | | | | |
| Difference between means | 7.2 | | | | |
| SE of difference | 3.867 | | | | |
| 95% CI of difference | -1.094 to 15.50 | | | | |
| | | | | | |
| **Bonferroni's multiple comparisons test** | **Mean Diff.** | **95.00% CI of diff.** | **Significant?** | **Summary** | **Adjusted P Value** |
| IS - IR+IS | | | | | |
| 0-25 | 5.831 | -5.335 to 17.00 | No | ns | 0.7326 |
| 25-50 | 7.681 | -3.485 to 18.85 | No | ns | 0.325 |
| 50-100 | 7.724 | -3.442 to 18.89 | No | ns | 0.3185 |
| 100-200 | 7.566 | -3.599 to 18.73 | No | ns | 0.3431 |

IS = ischemic stroke, IR+IS = irradiation+stroke

## Supplementary Table S3: ANOVA of DCX cell counts in cortex

| | | | | | | |
|---|---|---|---|---|---|---|
| **ANOVA summary** | | | | | | |
| F | 73.38 | | | | | |
| P value | <0.0001 | | | | | |
| P value summary | **** | | | | | |
| Significant diff. among means (P < 0.05)? | Yes | | | | | |
| R squared | 0.8303 | | | | | |
| | | | | | | |
| **Brown-Forsythe test** | | | | | | |
| F (DFn, DFd) | 15.28 (3, 45) | | | | | |
| P value | <0.0001 | | | | | |
| P value summary | **** | | | | | |
| Are SDs significantly different (P < 0.05)? | Yes | | | | | |
| | | | | | | |
| **Bartlett's test** | | | | | | |
| Bartlett's statistic (corrected) | 147.3 | | | | | |
| P value | <0.0001 | | | | | |
| P value summary | **** | | | | | |
| Are SDs significantly different (P < 0.05)? | Yes | | | | | |
| | | | | | | |
| **ANOVA table** | **SS** | **DF** | **MS** | **F (DFn, DFd)** | **P value** | |
| Treatment (between columns) | 2315928 | 3 | 771976 | F (3, 45) = 73.38 | P<0.0001 | |
| Residual (within columns) | 473415 | 45 | 10520 | | | |
| Total | 2789343 | 48 | | | | |
| | | | | | | |
| **Bonferroni's multiple comparisons test** | **Mean Diff.** | **95.00% CI of diff.** | **Significant?** | **Summary** | **Adjusted P Value** | |
| Ctrl vs. IR | 8.18 | -107.4 to 123.7 | No | ns | >0.9999 | |
| Ctrl vs. IS | -547.1 | -659.5 to -434.7 | Yes | **** | <0.0001 | |
| Ctrl vs. IR+IS | -178.3 | -285.6 to -71.07 | Yes | *** | 0.0002 | |
| IR vs. IS | -555.3 | -679.0 to -431.6 | Yes | **** | <0.0001 | |
| IR vs. IR+IS | -186.5 | -305.6 to -67.44 | Yes | *** | 0.0005 | |
| IS vs. IR+IS | 368.8 | 252.8 to 484.7 | Yes | **** | <0.0001 | |

Ctrl = control group, IR = irradiation, IS = ischemic stroke, IR+IS = irradiation+stroke

## Supplementary Table S4: ANOVA of DCX cell counts in Striatum 1

| | | | | | | |
|---|---|---|---|---|---|---|
| **ANOVA summary** | | | | | | |
| F | 39.68 | | | | | |
| P value | <0.0001 | | | | | |
| P value summary | **** | | | | | |
| Significant diff. among means (P < 0.05)? | Yes | | | | | |
| R squared | 0.7301 | | | | | |
| | | | | | | |
| **Brown-Forsythe test** | | | | | | |
| F (DFn, DFd) | 2.080 (3, 44) | | | | | |
| P value | 0.1166 | | | | | |
| P value summary | ns | | | | | |
| Are SDs significantly different (P < 0.05)? | No | | | | | |
| | | | | | | |
| **Bartlett's test** | | | | | | |
| Bartlett's statistic (corrected) | 8.229 | | | | | |
| P value | 0.0415 | | | | | |
| P value summary | * | | | | | |
| Are SDs significantly different (P < 0.05)? | Yes | | | | | |
| | | | | | | |
| **ANOVA table** | **SS** | **DF** | **MS** | **F (DFn, DFd)** | **P value** | |
| Treatment (between columns) | 11546012 | 3 | 3848671 | F (3, 44) = 39.68 | P<0.0001 | |
| Residual (within columns) | 4268020 | 44 | 97000 | | | |
| Total | 15814032 | 47 | | | | |
| | | | | | | |
| **Bonferroni's multiple comparisons test** | **Mean Diff.** | **95.00% CI of diff.** | **Significant?** | **Summary** | **Adjusted P Value** | |
| Ctrl vs. IR | 735.8 | 373.0 to 1099 | Yes | **** | <0.0001 | |
| Ctrl vs. IS | -553.4 | -895.0 to -211.8 | Yes | *** | 0.0003 | |
| Ctrl vs. IR+IS | 606.5 | 280.5 to 932.6 | Yes | **** | <0.0001 | |
| IR vs. IS | -1289 | -1676 to -902.4 | Yes | **** | <0.0001 | |
| IR vs. IR+IS | -129.3 | -502.4 to 243.9 | No | ns | >0.9999 | |
| IS vs. IR+IS | 1160 | 807.4 to 1512 | Yes | **** | <0.0001 | |

Ctrl = control group, IR = irradiation, IS = ischemic stroke, IR+IS = irradiation+stroke

## Supplementary Table S5: ANOVA of DCX cell counts in Striatum 2

| | | | | | |
|---|---|---|---|---|---|
| **ANOVA summary** | | | | | |
| F | 17.28 | | | | |
| P value | <0.0001 | | | | |
| P value summary | **** | | | | |
| Significant diff. among means (P < 0.05)? | Yes | | | | |
| R squared | 0.5525 | | | | |
| | | | | | |
| **Brown-Forsythe test** | | | | | |
| F (DFn, DFd) | 6.545 (3, 42) | | | | |
| P value | 0.001 | | | | |
| P value summary | *** | | | | |
| Are SDs significantly different (P < 0.05)? | Yes | | | | |
| | | | | | |
| **Bartlett's test** | | | | | |
| Bartlett's statistic (corrected) | 45.57 | | | | |
| P value | <0.0001 | | | | |
| P value summary | **** | | | | |
| Are SDs significantly different (P < 0.05)? | Yes | | | | |
| | | | | | |
| **ANOVA table** | **SS** | **DF** | **MS** | **F (DFn, DFd)** | **P value** |
| Treatment (between columns) | 60348 | 3 | 20116 | F (3, 42) = 17.28 | P<0.0001 |
| Residual (within columns) | 48883 | 42 | 1164 | | |
| Total | 109231 | 45 | | | |
| | | | | | |
| **Bonferroni's multiple comparisons test** | **Mean Diff.** | **95.00% CI of diff.** | **Significant?** | **Summary** | **Adjusted P Value** |
| Ctrl vs. IR | 48.4 | 8.569 to 88.23 | Yes | ** | 0.0099 |
| Ctrl vs. IS | -31.23 | -68.73 to 6.273 | No | ns | 0.1568 |
| Ctrl vs. IR+IS | 61.27 | 23.77 to 98.77 | Yes | *** | 0.0003 |
| IR vs. IS | -79.63 | -122.1 to -37.17 | Yes | **** | <0.0001 |
| IR vs. IR+IS | 12.87 | -29.59 to 55.33 | No | ns | >0.9999 |
| IS vs. IR+IS | 92.5 | 52.22 to 132.8 | Yes | **** | <0.0001 |

Ctrl = control group, IR = irradiation, IS = ischemic stroke, IR+IS = irradiation+stroke

## Supplementary Table S6: ANOVA of Iba1 cell counts

| | | | | | | |
|---|---|---|---|---|---|---|
| **ANOVA summary** | | | | | | |
| F | 120.7 | | | | | |
| P value | <0.0001 | | | | | |
| P value summary | **** | | | | | |
| Significant diff. among means (P < 0.05)? | Yes | | | | | |
| R squared | 0.9282 | | | | | |
| | | | | | | |
| **Brown-Forsythe test** | | | | | | |
| F (DFn, DFd) | 4.208 (3, 28) | | | | | |
| P value | 0.0141 | | | | | |
| P value summary | * | | | | | |
| Are SDs significantly different (P < 0.05)? | Yes | | | | | |
| | | | | | | |
| **Bartlett's test** | | | | | | |
| Bartlett's statistic (corrected) | 24.52 | | | | | |
| P value | <0.0001 | | | | | |
| P value summary | **** | | | | | |
| Are SDs significantly different (P < 0.05)? | Yes | | | | | |
| | | | | | | |
| **ANOVA table** | **SS** | **DF** | **MS** | **F (DFn, DFd)** | **P value** | |
| Treatment (between columns) | 588489 | 3 | 196163 | F (3, 28) = 120.7 | P<0.0001 | |
| Residual (within columns) | 45490 | 28 | 1625 | | | |
| Total | 633978 | 31 | | | | |
| | | | | | | |
| **Bonferroni's multiple comparisons test** | **Mean Diff.** | **95.00% CI of diff.** | **Significant?** | **Summary** | **Adjusted P Value** | |
| Ctrl vs. IR | 28.29 | -28.92 to 85.51 | No | ns | >0.9999 | |
| Ctrl vs. IS | -314.9 | -372.1 to -257.6 | Yes | **** | <0.0001 | |
| Ctrl vs. IR+IS | -132 | -189.2 to -74.77 | Yes | **** | <0.0001 | |
| IR vs. IS | -343.2 | -400.4 to -285.9 | Yes | **** | <0.0001 | |
| IR vs. IR+IS | -160.3 | -217.5 to -103.1 | Yes | **** | <0.0001 | |
| IS vs. IR+IS | 182.9 | 125.7 to 240.1 | Yes | **** | <0.0001 | |

Ctrl = control group, IR = irradiation, IS = ischemic stroke, IR+IS = irradiation+stroke

## Supplementary Table S7: ANOVA of % CD68/Iba1 cells

| | | | | | | |
|---|---|---|---|---|---|---|
| **ANOVA summary** | | | | | | |
| F | 12.2 | | | | | |
| P value | <0.0001 | | | | | |
| P value summary | **** | | | | | |
| Significant diff. among means (P < 0.05)? | Yes | | | | | |
| R squared | 0.428 | | | | | |
| | | | | | | |
| **Brown-Forsythe test** | | | | | | |
| F (DFn, DFd) | 6.32 (3, 49) | | | | | |
| P value | 0.001 | | | | | |
| P value summary | ** | | | | | |
| Are SDs significantly different (P < 0.05)? | Yes | | | | | |
| | | | | | | |
| **Bartlett's test** | | | | | | |
| Bartlett's statistic (corrected) | 75.2 | | | | | |
| P value | <0.0001 | | | | | |
| P value summary | **** | | | | | |
| Are SDs significantly different (P < 0.05)? | Yes | | | | | |
| | | | | | | |
| **ANOVA table** | **SS** | **DF** | **MS** | **F (DFn, DFd)** | **P value** | |
| Treatment (between columns) | 7045 | 3 | 2348 | F (3, 49) = 12.2 | P<0.0001 | |
| Residual (within columns) | 9434 | 49 | 193 | | | |
| Total | 16480 | 52 | | | | |
| | | | | | | |
| **Bonferroni's multiple comparisons test** | **Mean Diff.** | **95.00% CI of diff.** | **Significant?** | **Summary** | **Adjusted P Value** | |
| Ctrl vs. IR | 1.67 | -13.9 to 17.2 | No | ns | >0.9999 | |
| Ctrl vs. IS | -24.6 | -38.5 to -10.6 | Yes | **** | <0.0001 | |
| Ctrl vs. IR+IS | -19.2 | -33.6 to -4.71 | Yes | ** | 0.0039 | |
| IR vs. IS | -26.2 | -41.8 to -10.7 | Yes | *** | 0.0002 | |
| IR vs. IR+IS | -20.8 | -36.9 to -4.78 | Yes | ** | 0.0049 | |
| IS vs. IR+IS | 5.41 | -9.04 to 19.9 | No | ns | >0.9999 | |

Ctrl = control group, IR = irradiation, IS = ischemic stroke, IR+IS = irradiation+stroke
